# Human-to-mosquito transmission efficiency increases as malaria is controlled

**DOI:** 10.1038/ncomms7054

**Published:** 2015-01-19

**Authors:** Thomas S. Churcher, Jean-François Trape, Anna Cohuet

**Affiliations:** 1 Department of Infectious Disease Epidemiology, MRC Centre for Outbreak Analysis and Modelling, Imperial College London, London W2 1PG, UK; 2 Maladies Infectieuses et Tropicales Emergentes (URMITE) CNRS-IRD 198 UMR 6236, Institut de Recherche pour le Développement, BP 1386 Dakar, Sénégal; 3 Institut de Recherche en Sciences de la Santé, 399 Avenue de la Liberté, Bobo-Dioulasso 01 BP 545, Burkina Faso; 4 Maladies Infectieuses et Vecteurs, Ecologie, Génétique, Evolution et Contrôle (MIVEGEC) UM1-UM2-CNRS 5290-IRD 224, Institut de Recherche pour le Développement, BP 64501, 34394 Montpellier Cedex 5, France

## Abstract

The efficiency of malaria transmission between human and mosquito has been shown to be influenced by many factors in the laboratory, although their impact in the field and how this changes with disease endemicity are unknown. Here we estimate how human–mosquito transmission changed as malaria was controlled in Dielmo, Senegal. Mathematical models were fit to data collected between 1990 and the start of vector control in 2008. Results show that asexual parasite slide prevalence in humans has reduced from 70 to 20%, but that the proportion of infectious mosquitoes has remained roughly constant. Evidence suggests that this is due to an increase in transmission efficiency caused by a rise in gametocyte densities, although the uneven distribution of mosquito bites between hosts could also contribute. The resilience of mosquito infection to changes in endemicity will have important implications for planning disease control, and the development and deployment of transmission-reducing interventions.

## Abstract (web summary)

Understanding the epidemiology of malaria transmission between humans and mosquitoes is crucial for successful disease control. Analysing data from an 18-year malaria control programme, Churcher *et al*. show that decreased parasite prevalence in humans can be found concurrently with an increase in transmission efficiency.

## Graphical abstract

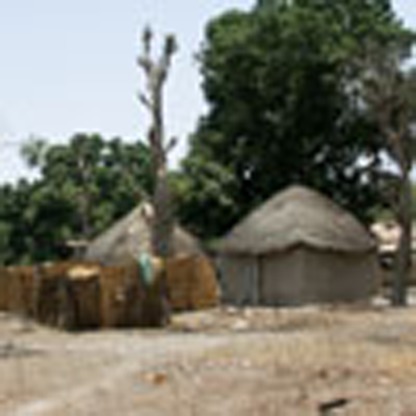

The processes governing malaria transmission are diverse and complex. Human–mosquito transmission occurs when blood-feeding mosquitoes ingest parasite transmission stages (gametocytes) circulating in the peripheral blood. The parasite undergoes sexual reproduction before invading the midgut and developing into oocysts and ultimately sporozoites, which migrate to salivary glands and are capable of onwards transmission. Mosquito-feeding experiments have shown that a wide range of host, parasite and mosquito factors influence the likelihood that a mosquito will acquire the infection. These include hosts anaemia^1^, immunity^2^ and drug treatment^3^; parasite (gametocyte) density^4^, maturity^5^ and clone diversity^6^; and mosquito size^7^, microbial gut flora^8^, environment^9^ and immune response^10^ among others. These explanatory variables are rarely collectively measured, making it difficult to predict how transmission efficiency changes between locations and following the introduction of control interventions. It is also unclear how transmission may differ between natural infections and those conducted with laboratory-adapted mosquitoes in controlled conditions^11^, making it difficult to predict the impact of these different factors in field settings.

Parallel human and entomological surveys provide an alternative method of quantifying human–mosquito transmission in natural settings. More efficient transmission represents more mosquitoes being infected at a given endemicity. These data are routinely collected to assess malaria prevalence and to calculate the entomological inoculation rate (EIR, the average number of infectious bites per person per year). Most data sets are cross-sectional, which restricts their use for determining transmission efficiency, as parameters determining sporozoite prevalence are likely to vary from location to location. For example, the proportion of infectious mosquitoes depends upon both the transmission efficiency, but also on the length of the extrinsic incubation period, and the mosquito death rate^12^. Both of these factors are likely to vary between sites^13^,^14^. There may also be a difference in strains of the parasite and mosquito, which may influence vector competence^11^. All these processes will themselves change endemicity, which makes it difficult to ascribe changes in the relationship between human and mosquito prevalence to changes in transmission efficiency. Many of these problems can be overcome using longitudinal studies, where the biology and epidemiology of the parasite and vector are likely to be more consistent. Diagnostic sensitivity and mosquito-trapping techniques are also likely to be less variable increasing the precision of results.

The Dielmo Project in Senegal started in 1990 and has regularly estimated human and mosquito prevalence in the same village for the last 23 years^15^,^16^. These data provide a unique data set to test whether human–mosquito transmission efficiency has changed as malaria has been controlled. A number of factors make this site particularly amenable to this type of analysis. First, the mosquito age distribution (as measured by parity) has remained roughly constant between 1990 and the introduction of insecticide-treated nets in July 2008. During this 18-year period ~50% of the population used untreated bednets^16^. Although these may have some impact on mosquito mortality^17^, the consistent age distribution makes it unlikely that any variability in sporozoite prevalence is caused by changes in mosquito survival. Vector density has also remained broadly the same over the period, although there is some variability between years. If it is assumed that the length of the extrinsic incubation period has remained constant over time (as annual temperatures have remained broadly the same^18^), then it seems reasonable to ascribe changes in the relationship between human and mosquito infection to changes in the human–mosquito transmission probability. Second, good clinical management of cases has reduced malaria endemicity (as measured by asexual parasite slide prevalence, subsequently referred to as prevalence) substantially^16^. In 1990, malaria prevalence in the village was 68%; in 2007, this had reduced to 30%. The gradual reduction allows the investigation of how transmission efficacy changes as the disease is progressively controlled. Third, malaria prevalence is relatively consistent throughout the year. Seasonality in transmission increases the variability in the sporozoite prevalence, so changes in human–mosquito transmission will be easier to detect in perennial sites. Although there are seasonal fluctuations in mosquito abundance, their regularity and the consistent timing of data collection should reduce variability and bias. Lastly, the presence of two different groups of vectors *Anopheles gambiae sensu lato* and *Anopheles funestus* allows two estimates of the transmission probability and facilitates a comparison between species.

Transmission reduction is an integral part of efforts to control and eliminate malaria^19^. Understanding how human–mosquito transmission changes as the disease is successfully controlled will be essential to plan interventions accurately and evaluate the public health impact of transmission-reducing drugs and vaccines. Here we fit a suite of simple mathematical models to parallel human and entomological data to estimate how transmission efficiency changes with malaria prevalence in the village of Dielmo between 1990 and 2008. Estimates of human and mosquito infection prevalence are uncertain, so Bayesian techniques are used to accurately account for measurement error and statistically determine whether human–mosquito transmission changes with malaria prevalence. Results indicate that as malaria has been successfully controlled transmission efficiency has increased, possibly through an increase in gametocyte density in infected individuals.

## Results

### Temporal trends

**Figure 1:**
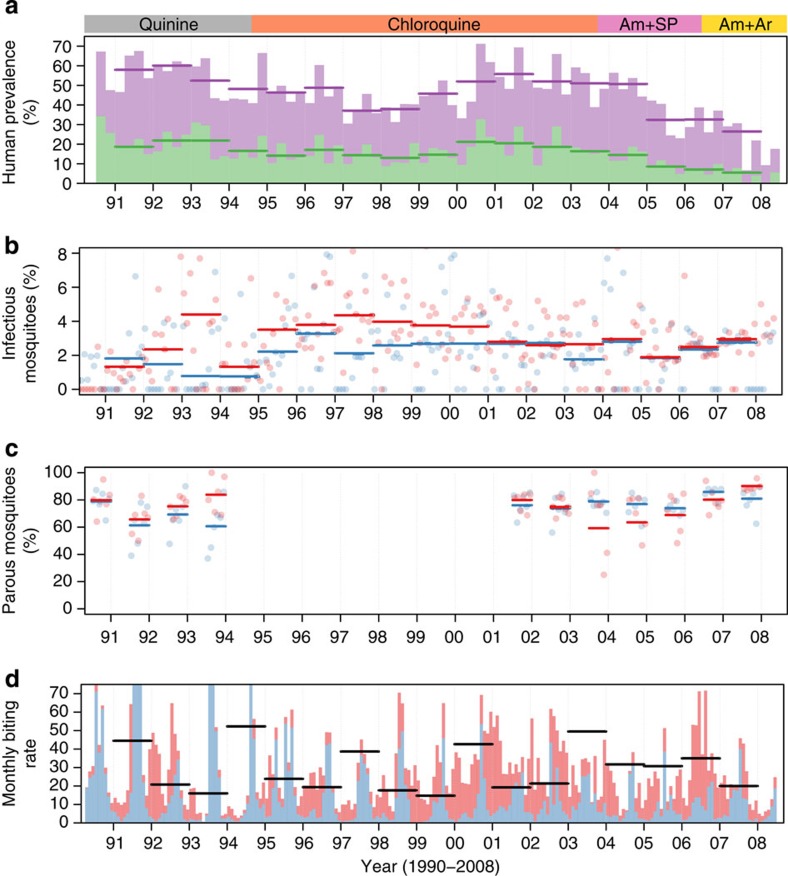
Summary of the Dielmo data between 1990 and the start of vector control in July 2008. (**a**) The changes in *Plasmodium falciparum* prevalence of asexual parasites (purple) and gametocytes (green) in the enrolled population as measured by microscopy. (**b**) The percentage of *Anopheles gambiae s.l.* (blue) and *A. funestus* (red) with salivary gland sporozoites. (**c**) The parity of *A. gambiae s.l.* (blue) and *A. funestus* (red) (data were only available for certain months between 1990–1994 and 2001–2008, see Methods). (**d**) The cumulative monthly biting rate of *A. gambiae s.l.* (blue) and *A. funestus* (red). In all panels horizontal lines show the mean over the years (lines are omitted when there is missing data for the period). The changes in the drugs used for first-line therapy are shown at the top of the figure, be it quinine (grey), chloroquine (orange), amodiaquine plus sulphadoxine-pyrimethamine (Am+SP, pink) or amodiaquine plus artesunate (Am+Ar, yellow). In addition to the treatment of cases, there was no other use of antimalarials but for intermittent preventive treatment for pregnant women (one dose of SP quarterly from 2004).

Malaria prevalence decreases significantly between 1990 and 2008 (Fig. 1a). During this time, the proportion of infectious mosquitoes either remains constant (*A. funestus*) or increases slightly (*A. gambiae*, Fig. 1b). Gametocyte slide prevalence also significantly decreases (Fig. 1a) as does the ratio of gametocyte prevalence to asexual parasite prevalence (Supplementary Fig. 1). The size and age distribution of the mosquito population (as measured by parity) remains roughly constant (Fig. 1c,d), suggesting that human–mosquito transmission has become increasingly efficient to enable the high level of mosquito infection to be maintained. Simple statistical analyses of the broad temporal trends are presented in Supplementary Fig. 1.

### Changes in human–mosquito transmission

**Figure 2:**
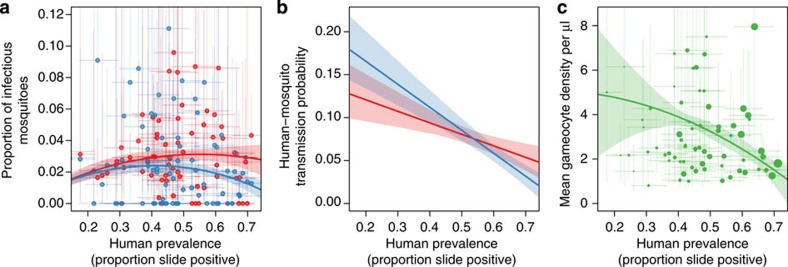
Best-fit models. (**a**) The relationship between the proportion of mosquitoes with salivary gland sporozoites and the prevalence of asexual parasites in the human population. Each point shows human and entomological surveys conducted during the same month. Blue points are *Anopheles gambae s.l.* and red *A. funestus*. Thick lines give the best-fit model for each mosquito species. (**b**) The change in the human–mosquito transmission efficiency (expressed as a proportion) with the proportion of asexual parasites in the human population. Blue line is for *A. gambae s.l.* and red for *A. funestus.* (**c**) The relationship between the mean gametocyte density in gametocyte-positive patients (per microlitre of blood, as detected by microscopy) and the proportion of patients with asexual parasites. In all plots horizontal and vertical lines denote the 95% confidence intervals around point estimates while the thick solid lines and shaded areas show the median and the 95% Bayesian credibility intervals for the posterior distribution, respectively.

To statistically determine whether transmission efficiency varies with prevalence, a set of nested mathematical models were fit to parallel human and mosquito infection data. Allowing human–mosquito transmission probability to change with asexual parasites prevalence (as measured by microscopy) significantly improved model fit (Supplementary Table 1). Different functional forms for the relationship between prevalence and transmission probability were statistically compared, with a linear relationship that allowed the two mosquito species to have different transmission probabilities giving the most parsimonious fit. The best-fit model indicates that when 70% of villagers have slide-detectable asexual parasites, ~5% of mosquitoes biting an infected host will develop sporozoites. Reducing malaria prevalence to 20% increases the transmission probability to ~14% (Fig. 2a,b). A similar pattern is seen in both *A. gambiae* and *A. funestus*, indicating that the result is consistent across mosquito species. The analysis was repeated using the prevalence of gametocyte (transmission stages) instead of prevalence of asexual parasites. Again a similar pattern was observed, with models that allow transmission efficacy to change with gametocyte prevalence giving significantly better fits (Supplementary Table 1). The increase in transmission efficiency could be explained in part by an increase in the number of gametocytes in patients where they were detectable by microscopy (Fig. 2c). In gametocyte-positive hosts, the density of gametocytes doubled as asexual parasite slide prevalence dropped from 70 to 40%. A variety of frontline therapies have been used over the years, starting with quinine before switching to chloroquine (1994), amodiaquine plus sulphadoxine-pyrimethamine (Am+Sp, 2003) and finally amodiaquine plus artesunate (Am+Ar, 2006). Chloroquine treatment has been shown to induce gametocytogenesis and increase mosquito infection^20^, particularly in areas where the parasite has developed drug resistance^21^. To see whether the change in transmission probability and gametocyte density was independent of the frontline therapy, the analysis was repeated for each treatment regimen separately. Results are consistent across all the four treatment periods, with each showing increasing human–mosquito transmission and gametocyte density as malaria prevalence falls (Supplementary Fig. 2). The model that allowed vector mortality to change relative to one another did not improve the fit, suggesting that the baseline parameters were sufficiently accurate.

**Figure 3:**
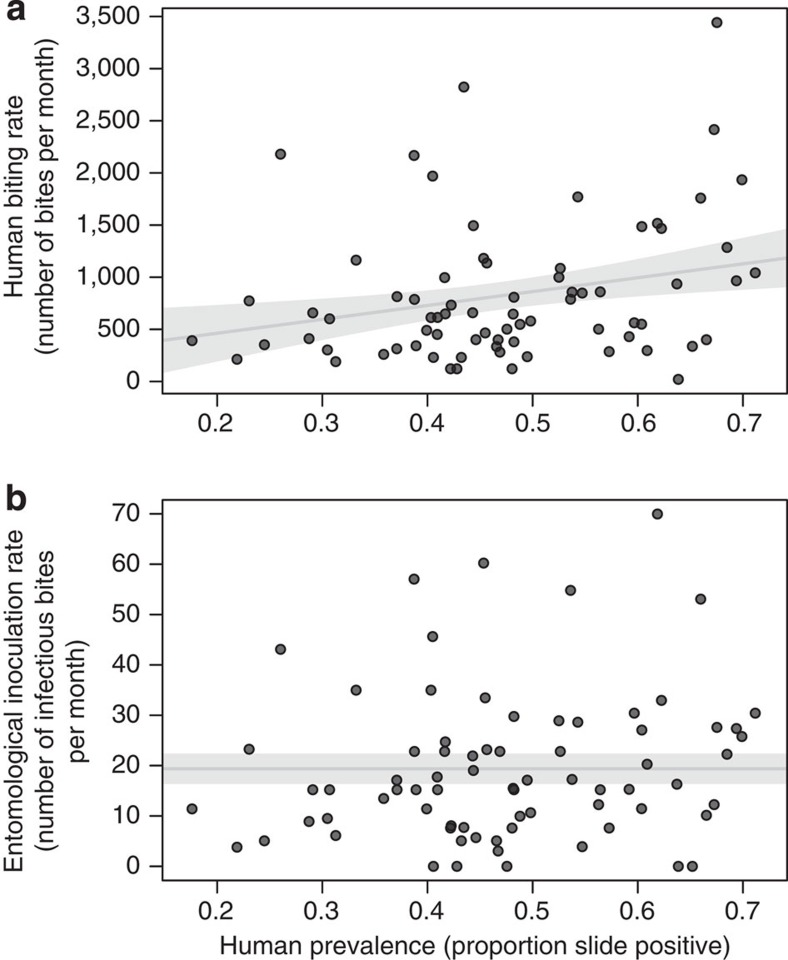
The relationship between entomological measurements and the proportion of the population with asexual parasites. (**a**) The changes in the average number of mosquito bites per person over a month (human biting rate) and the proportion of asexual parasites. (**b**) The relationship between the average number of infectious bites received per person per month (the entomological inoculation rate, EIR) with malaria asexual parasite slide prevalence. In both panels, the solid grey line shows the results of the best-fit linear regression, which was used to test for an association between the two measurements. The 76 data points showed a significant association between prevalence and the biting rate (estimated by human landing catches, *P*=0.013), although there was no evidence of a relationship between malaria prevalence and EIR (*P*=0.172). Closed dots give the individual point estimates while the shaded area indicates the 95% confidence intervals around the best-fit linear regression.

The change in transmission efficiency complicates the relationship between disease prevalence and the human force of infection (as measured by EIR). Linear regression is unable to detect a significant association between prevalence and EIR, either with all data analysed together or separately according to frontline treatment (Fig. 3). There was a positive correlation between monthly human biting rates and prevalence (Fig. 3a), which appears to counteract the observed changes in the human–mosquito transmission probability so that there is no discernible change in EIR with prevalence (Fig. 3b).

### Heterogeneous biting

**Figure 4:**
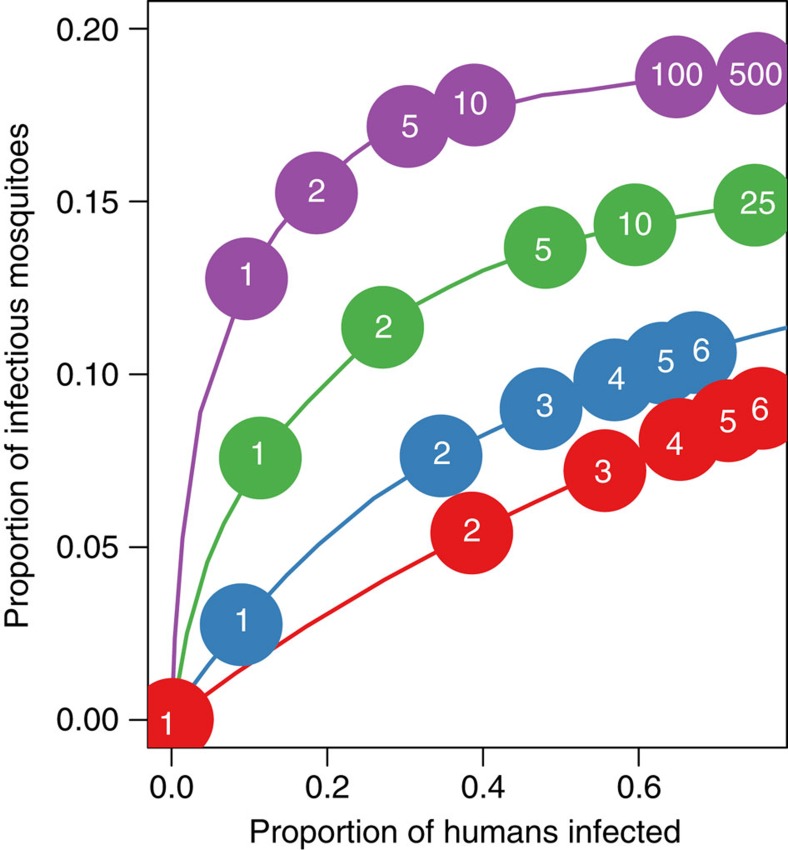
An illustration of how biting heterogeneity influences the relationship between human and mosquito infection. The figure presents the results of a simple mathematical model that shows how the uneven biting of mosquitoes between hosts influences the proportion of infectious mosquitoes and humans at endemic equilibrium. Biting heterogeneity is investigated by varying the percentage of total bites that are taken on the 20% of the people who are bitten the most, be it 20% (that is, homogenous biting, *ν*=0, red line), 40% (*ν*=0.405, blue line), 60% (*ν*=1.55, green line) or 80% (*ν*=4.10, purple line). The points and the white numbers within them show the model-derived basic reproduction number, *R*_0_, required to generate that endemic equilibrium. In addition to the parameter values outlined in the main text *b*=0.05, *c*=0.2, and the mosquito is assumed to be *An. funestus*. Care should be taken when interpreting this figure as the mathematical model is used as an illustration and is not specifically parameterized for Dielmo.

A possible cause for the relationship between human and mosquito infection could be the uneven distribution of mosquito bites across the human population (often referred to as heterogeneous biting or hotspots of transmission). Evidence suggests that in some locations a core group of the human population receive a substantial proportion of mosquito bites (for example, up to 80% of the bites might be taken on just 20% of the population^22^). This core group will have a higher probability of having malaria and is more likely to remain infected if prevalence in the overall population falls. The majority of mosquitoes will be infected by the core group, so although the prevalence of human infection outside the core group may go down, these hosts are bitten relatively rarely so they will have a smaller impact on the overall proportion of infected mosquitoes. Mosquito infection will therefore decline at a slower rate the more heterogeneous the biting is. The phenomenon is illustrated with a simple mathematical model presented in Fig. 4. This suggests that there needs to be substantial biting heterogeneity for mosquito infection to remain constant for the range of endemicities observed in Dielmo (that is, >80% of the bites might be taken on 20% of hosts). The basic reproductive number, *R*_0_, is defined as the average number of infections caused by an infection in an uninfected population, and is widely used as a measure of malaria transmission intensity^12^. Figure 4 illustrates that in areas with high biting heterogeneity a very high *R*_0_ is required to achieve a slide prevalence of 70% observed at the start of the study. This is because most of the mosquito bites are concentrated on the core group, so there must be very efficient transmission (for example, a high number of mosquitoes) for those who are bitten relatively rarely to become infected. The simple model indicates that at the highest level of biting heterogeneity (80% of bites on 20% of hosts) there must have been a 99.6% reduction in *R*_0_ over the course of the study for slide prevalence to have dropped from 70 to 20%. For lower levels of biting heterogeneity (40% of bites on 20% of hosts) this reduces to a ~80% reduction in *R*_0_.

It is unclear exactly what the degree of biting heterogeneity is in Dielmo, as no specific study to address this has been carried out. The small geographical area, homogeneity in type of housing and the permanent river running beside the village suggest that heterogeneity may be relatively minimal. A generalized linear mixed model (GLMM) was fit to the human data to determine whether some hosts were slide positive more than others. Examination of the individual random-effects estimates of the model suggest that there is relatively minimal heterogeneity in the probability of being slide positive (with the s.d. of the intercept being 0.86, Supplementary Fig. 3). As an illustration, the model predicts that in the 10- to 14-year age group in the last quarter of 2007 when malaria prevalence is 23%, the 20% of hosts who are the most likely to be infected accounted for only 37% of slide-positive infections (Supplementary Fig. 3).

## Discussion

Transmission between human and mosquito is a dynamic process that may become more efficient as malaria is successfully controlled. There are many possible reasons for this trend^1^,^2^,^3^,^4^,^5^,^6^,^7^,^8^,^9^,^10^, although the data suggest that it may be in part caused by an increase in gametocyte density in gametocyte-positive patients. Over the course of the study, the increase in gametocyte density is comparable to the increase in transmission efficiency predicted by the model. However, this association does not prove causality, and other processes could have contributed to the observed result. Prevalence declines over the study period, so any temporal change in human, parasite or mosquito factors that influences human–mosquito transmission could have caused the increase in transmission.

The analysis suggests that the transmission dynamics of *A. gambiae s.l.* and *A. funestus* are slightly different, with models that allow the efficiency of transmission to vary at different rates giving the most parsimonious fit. The similarity of the lines suggests that the change in transmission efficiency is driven by host factors, not vector competence. Nevertheless, secular changes to the local climate and environment could influence both species and therefore cannot be discounted. A wide range of factors have been shown to increase mosquito longevity, which would increase the number of infectious mosquitoes. Although the parity data are incomplete, there does not appear to be a substantial change in the age distribution between 2001 and 2008, the time that sees the greatest fall in prevalence (Fig. 1; Supplementary Fig. 1). Parity is an imperfect measure of mosquito survival^23^, although it is unlikely to mask the considerable increase in longevity required to explain the observed sprozoite prevalence. For example, for *A. funestus*, equation (1) indicates that mean mosquito life-expectancy would need to be increased by >1/3 (from 8.9 to 12.2 days) as prevalence dropped from 70 to 30%, if the transmission probability remained constant at 0.06. Temporal changes in mosquito susceptibility over time could also be a possibility as could an increase in mosquitoes size caused by changing larval conditions. However, this would also likely be evident in changes in mosquito longevity (as larger mosquitoes live for longer^24^), and there has been no discernible change in local climatic conditions^16^. Another hypothesis, which could explain the high mosquito infection rates seen at low malaria prevalence, might be that infected mosquitoes were migrating into the village from the surrounding area having fed on people with a higher malaria prevalence. This is unlikely to be the case since Dielmo is relatively geographically isolated, and the permanent river provides sufficient breeding sites for mosquitoes, which will reduce their geographical range^15^.

Some of the apparent change in human–mosquito transmission could be ascribed to microscopy becoming increasingly inefficient at detecting infections as prevalence falls^25^. If this was the case then the true drop in malaria prevalence would be less pronounced, and transmission efficiency would not need to increase so much to generate the observed sporozoite prevalence. It has been suggested that at a slide prevalence of 20%, up to 25% of the infectious reservoir might reside in slide-negative individuals^25^. Such an underestimation would be insufficient to describe the observed patterns, indicating that some increase in transmission efficacy has been observed.

The model structure used was relatively simple and assumes that everyone is bitten equally. In reality, biting is likely to be more heterogeneous, with some people being bitten more than others. This has been proposed to explain why mosquito–human transmission becomes less efficient in areas of higher endemicity^26^. A simple mathematical model is used to show that heterogeneous biting does make the relationship between human and mosquito infection increasingly nonlinear. This model is not specifically parameterized for Dielmo, although it suggests that for heterogeneous biting to be the sole cause, more than 80% of mosquito bites will need to have been taken on 20% of the residents. Given the geography of the village and proximity of breeding sites, this extreme heterogeneity appears unlikely. This is backed up by the longitudinal data, which suggesting that, although some people are more likely to have detectable asexual parasites than others, the degree of heterogeneity is relatively low (although the heterogeneity in malaria-positive slides may be less pronounced than the heterogeneity in mosquito biting due to human superinfection). In addition, the rapid decline in malaria prevalence caused by drug treatment makes it unlikely that heterogeneity in biting alone could have caused the persistence of mosquito infection. Locations with high biting heterogeneity would require a very high reduction in *R*_0_ for prevalence to drop from 70 to 20% (as a very high *R*_0_ is needed to achieve a high prevalence). Mosquito density has remained roughly constant over the period, so the observed decline in malaria prevalence is most likely caused by a reduction in the duration of human infectiousness^16^. The simple model predicts that areas with the highest biting heterogeneity *R*_0_ would have to have decreased by >99% over the course of the study. This equates to a decrease in the duration of infectiousness of >99%, which seems infeasible for an area where all cases of fever receive prompt treatment. In comparison, if there is no heterogeneity in biting then a 70% reduction in duration of infectiousness would be sufficient, which is more in line with the changes seen following switching drug regimens in clinical trials^3^. Further work is required, although it seems likely that multiple factors including an increase in gametocyte density and heterogenenous biting contribute to the relationship between human and mosquito infection.

If the increase in transmission efficiency is caused by an increase in gametocyte density, then the reason for this is far from clear. It has been proposed that gametocyte concentration might be regulated by cross-stage immunity, either through reducing asexual parasite densities (which go on to produce gametocytes) or through the removal of circulating gametocytes directly^27^,^28^. There is also evidence that gametocyte-infected erythrocytes elicit a specific immune response, which has been associated with lower gametocyte densities^29^. This could explain why gametocyte carriage is longer in children than it is in adults^30^, and why the density of gametocytes relative to the total parasite concentration increases with age^31^. As malaria is successfully controlled and endemicity falls, the strength of immunity may wane and cause the observed increase in gametocyte density. Indeed, gametocytemia has been seen to increase as the disease is successfully controlled in other locations^32^,^33^. For example, in Western New Guinea, insecticide spraying and mass drug administration in the 1950s reduced disease prevalence but more than doubled the percentage of infections, which had detectable gametocytes^32^. In the Garki Project, Nigeria gametocyte prevalence increased in older residents to as much as double their baseline levels after control interventions were halted^34^. There was also an increase in gametocyte and asexual parasite density in patients with detectable malaria^34^. Both of these observations are consistent with the hypothesis that the increase in transmission efficiency could be due to higher gametocyte density. However, both studies indicated that the ratio of detectable gametocytes to asexual parasites went up as the disease was successfully controlled, which is the reverse of what was seen in Dielmo (Supplementary Fig. 1). This could be because in Dielmo all cases of fever are promptly treated, which is likely to impede the appearance of parasites in subsequent cross-sectional surveys.

The model suggests that at a given slide prevalence, the gametocyte density was higher when the community was treated with quinine or chloroquine than for Am+Sp or Am+Ar, which is in accordance with our current knowledge of drug action^35^. It cannot be discounted that the rise in chloroquine resistance over the period^36^ could have contributed to the increase in gametocyte density, although the similar patterns seen with other therapies suggest that it is not the sole reason.

Transmission-reducing immunity, which targets parasite sexual stages within the mosquito, has been shown to reduce gametocyte infectiousness^35^. These immune responses are thought to be relatively short-lived^37^ and therefore could also contribute to the increase in transmission efficiency seen as endemicity declines independent of any change in gametocyte density.

In highly endemic areas, mosquito–human transmission is thought to be relatively inefficient. As malaria prevalence falls, transmission dynamics and immunoepidemiology change and infected mosquitoes are more likely to transmit the infection^26^. This is an example of a negative frequency-dependent process that can have an important impact on parasite population dynamics. Here we show that human–mosquito transmission is also a negative frequency-dependent process that acts independently of any changes in efficiency of mosquito–human transmission. This could occur both at the individual level (due to changes in gametocyte density) and at the population level (due to heterogeneous biting). As malaria is successfully controlled and hyper-endemic regions become meso-endemic, human–mosquito transmission will on average become more efficient and the parasite is more likely to complete its lifecycle. This will have important epidemiological implications as it will increase the stability of disease and make it harder to control and eliminate. Current mathematical models of malaria tend to either assume a constant human–mosquito transmission parameter or one that changes as a function of asexual parasite density^38^,^39^. Although this may not influence equilibrium dynamics, with other parameters accounting for the changes in the transmission parameter, this oversimplification might cause the impact of control interventions to be overly optimistic.

Transmission reduction is increasingly seen as an important component of the disease elimination agenda^40^. There are currently a large number of transmission-reducing drugs and vaccines under development that aim to reduce human–mosquito transmission^35^. Many of these candidates are partially effective, reducing transmission from a host but not halting it. The lower efficiency of human–mosquito transmission in areas of higher malaria prevalence may make these interventions more efficacious^41^. This should be considered when decisions about where potential transmission-blocking vaccine candidates should be trialled, as it is currently thought that the interruption of transmission is more likely in areas of low transmission intensity^40^,^42^. Those planning the use of these interventions should be aware that transmission between man and mosquito might be a dynamic process and that their effectiveness may decrease as malaria is successfully controlled.

## Methods

### Data set

Parallel parasitological and entomological surveys from the Dielmo project, Senegal were collated between June 1990 and June 2008. A full description of the site and data collection methods is given elsewhere^15^,^16^. Briefly, all residents living in the village were invited to participate in a longitudinal study, which monitored the occurrence of fever daily. Each year >90% of villagers enrolled. Cross-sectional parasitological surveys using a think blood smear were carried out every quarter. A total of 200 high-powered fields were examined each time, which is assumed to correspond to ~0.5 μl blood^43^. Results showed that at baseline *Plasmodium falciparum*, *P. malariae* and *P. ovale* were endemic in Dielmo, with a baseline prevalence of 69.3%, 20.4% and 5.5%, respectively^16^. For simplicity only the *P. falciparum* data were analysed, as sporozoite prevalence estimates were not available for the other species. Human landing catches were conducted at the same time and place (two indoor and two outdoor) each month. The proportion of infected mosquitoes was measured by dissection of the salivary glands and epifluorescence (1990–1992) or by testing the *P. falciparum* circumsporozoite protein by enzyme-linked immunosorbent assay (1992–2008). Between 1990–1994 and 2001–2008 a subset of caught mosquitoes were dissected to determine parity (the proportion of females that had laid eggs). Matching concurrent human and entomological data (that is, conducted within the same month) gave a total of 76 time points. This comprising of a total of 26,544 blood smears, 26,152 mosquito dissections (46% *A. gambiae s.l.*) out of a total 27,283 mosquitoes caught (49% *A. gambiae s.l.*). An outline of the data set is given in Supplementary Table 2.

### Descriptive analysis

The temporal trends in human (asexual parasite and gametocyte) prevalence, mosquito prevalence, mosquito biting rate and parity are assessed using GLMMs. Models with and without time (since the start of the study, in years) as a fixed effect are compared using the likelihood ratio test. Month of data collection was included as a random effect to account for seasonality. A binomial error structure was used for prevalence and parity data while the number of mosquitoes caught in landing catches (averaged over person nights) was assumed to follow a normal distribution. Full parity data were only available from 2001 to 2008, so the analysis was restricted to this period. Point estimates of the percentage of parous mosquitoes were available from 1990 to 1995 and were included in the figures for illustrative purposes, although care should be taken as the number of mosquitoes dissected is likely to vary substantially. Ninety-five per cent confidence interval estimates were generated using the binomial distribution (exact method) for prevalence data while bootstrapping methodology was used for gametocyte density. Simple linear regression was used to determine whether the human biting rate or EIR changed with prevalence.

To assess whether some people were slide positive more than others, a GLMM was fit to human infection data assuming a binomial error structure (logit function) and adjusting for patient age and changes in prevalence over time. Person age (7 groups: 0–1, 2–4, 5–9, 10–14, 15–29, 30–44, ≥45 years) and month of survey were included as categorical variables. Host identification number was used as a random effect allowing the variability in the individual random-effect intercepts to give an indication of whether the same hosts are repeatedly slide positive. The model was then used to estimate what percentage of infections were detected in those hosts who were most infected over the course of the study.

### Mathematical model

A simple mathematical model is used to estimate human–mosquito transmission efficiency from survey data. Let *X*_*i*_ be the proportion of humans with microscopy detectable parasites at time point *i* (where *i*=1 .. 76). Although only gametocytes are infectious to mosquitoes, patients are initially considered positive with either asexual parasites or gametocytes since sub-microscopic gametocytes are likely to contribute to transmission^35^. The proportion of infectious mosquitoes, *Z*_*i*_, at time point *i* is given by the following equation^12^,


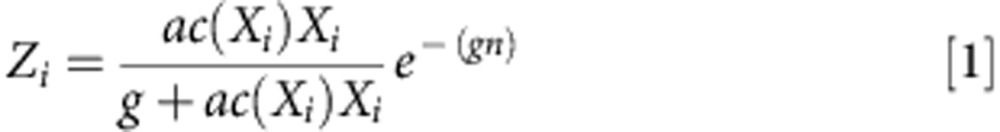


where *a* is the mosquito biting rate, *g* is the mosquito death rate and *n* is the mosquito latent period. Parameter *c*(*X*_*i*_) denotes the probability that a mosquito feeding on an infected host will develop salivary gland sporozoites (conditional on it surviving the latent period). To investigate whether transmission efficiency changes with asexual parasite slide prevalence, *c*(*X*_*i*_) is allowed to vary with *X*_*i*_ using the polynomial equation,


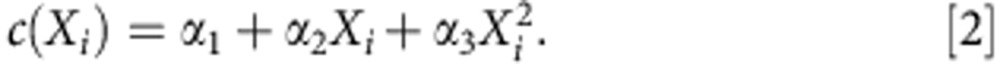


This flexible, nonlinear function allows for both increasing and decreasing values of *c* with increasing *X*_*i*_, and nests both a constant transmission probability (*α*_2_=*α*_3_=0) or one that varies linearly with slide prevalence (*α*_3_=0). It is assumed that mosquitoes bite every 3 days, die at a constant rate and live for an average of 7.6 days (*A. gambiae s.l.*) or 8.9 days (*A. funestus*)^38^,^44^. The latent period is taken to be 11 days.

### Model fitting

A suite of nested models are fit to the full human and mosquito data set and compared. Initially it is assumed that *c* is the same for *A. gambiae s.l.* and *A. funestus* mosquitoes. Next, it is allowed to vary between mosquito species generating species-specific rates of transmission. Finally, the mortality rate of *A. gambiae s.l.* is also allowed to vary to see whether different (although constant) mosquito life expectancies can describe the discrepancy between species. In all models the constant, linear and polynomial functions for *c*(*X*_*i*_) are tested.

All models were fit using Bayesian methods to account for the considerable measurement error. A binomial distribution is used to describe the malaria prevalence in both humans and mosquitoes. Numerical simulation was done using a Gibbs Markov chain Monte Carlo sampling algorithm implemented in OpenBUGS^45^. Three Markov chains were initialized to assess convergence and the first 5,000 Markov chain Monte Carlo iterations were discarded as burn in. A total of 10,000 iterations were used to derive the posterior distribution of parameters and generate 95% Bayesian credible interval estimates for model fits. Uniform priors *U*([−100,100]) were used for all parameters although parameter sets that gave negative values of *c* were excluded. Models were compared using the deviance information criterion^46^. Smaller values indicate a better fit, and a difference of five deviance information criterion units is considered to be substantial. To determine whether the effect was independent of drug regimen the analysis was repeated for each frontline therapy.

The relationship between gametocyte density (per 0.5 μl blood) in gametocytemic host and slide prevalence is estimated by fitting equation (2) to the individual gametocyte counts. A zero-truncated negative-binomial distribution is used to take into account the overdispersed nature of gametocyte count data with the overdispersion parameter allowed to vary with an uninformative prior *U*([0,10]).

### Heterogeneous biting transmission dynamics model

To illustrate how the uneven distribution of mosquito bites between hosts influences the relationship between human and mosquito infection, a simple transmission dynamics model of malaria^12^ is extended to incorporates biting heterogeneity. Following Smith *et al*.^26^, let *j* denote a group of patients who receive the same number of mosquito bites and *ω*_*j*_ be the relative number of bites they receive that is described by a gamma distribution with a population mean of one and a variance *ν*. If it is assumed that there is no site fidelity in mosquito biting (that is, the probability that a mosquito bites a host is not influenced by who they have bitten previously), then the change in *x*_*j*_(*t*), the proportion of infectious humans in group *j* at time *t*, and *z*_*j*_(*t*), the proportion of infectious mosquitoes biting on group *j* at time *t*, is given by the equations,


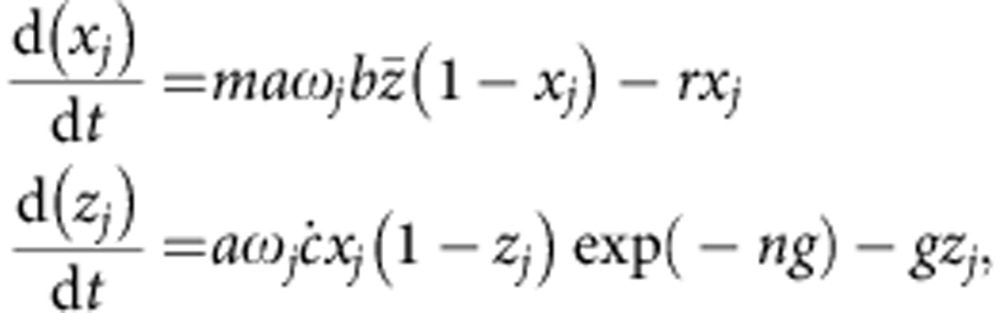


where *m* is the mosquito density per human, *b* denotes the mosquito-to-human transmission probability, *c* is the human-to-mosquito transmission probability (which here is assumed to be constant) and *r* is the human recovery rate. The mean infectivity of mosquitoes, 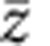, is weighted by how frequently people are bitten and is calculated using the equation, 
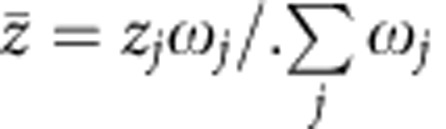
. Models are run until they reach endemic equilibrium initially assuming a mean duration of infectiousness of 50 days. Endemic equilibrium will vary according to the degree of biting heterogeneity (*ν*), so for each value of *ν* the mosquito density *m* is adjusted to enable human endemic prevalence to reach 70%. This value of *m* is then kept constant and the mean duration of infectiousness (1/*r*) is then decreased to generate the range of malaria prevalence’s observed in Diemlo. For each parameter set the basic reproduction number *R*_0_ is calculated using equations outlined in Smith and McKenzie^12^.

## Author contributions

T.S.C. and A.C. conceived the project, J.-F.T. collected the data and T.S.C. conducted the analysis and wrote the initial draft. All authors discussed the results and implications of the analysis and commented on the manuscript at all stages.

## Additional information

**How to cite this article:** Churcher, T. S. *et al*. Human to mosquito transmission efficiency increases as malaria is controlled. *Nat. Commun.* 6:6054 doi: 10.1038/ncomms7054 (2015).

## Supplementary Material

Supplementary Information Supplementary Figures 1-3 and Supplementary Tables 1-2
