# Supplementary material for: Human-to-mosquito transmission efficiency increases as malaria is controlled
Source: Nat Commun. 2015 Jan 19;6:6054. doi: 10.1038/ncomms7054 (PMC4309425; doi:10.1038/ncomms7054)
Supplement: Supplementary Information — Supplementary Figures 1-3 and Supplementary Tables 1-2 [file ncomms7054-s1.pdf]

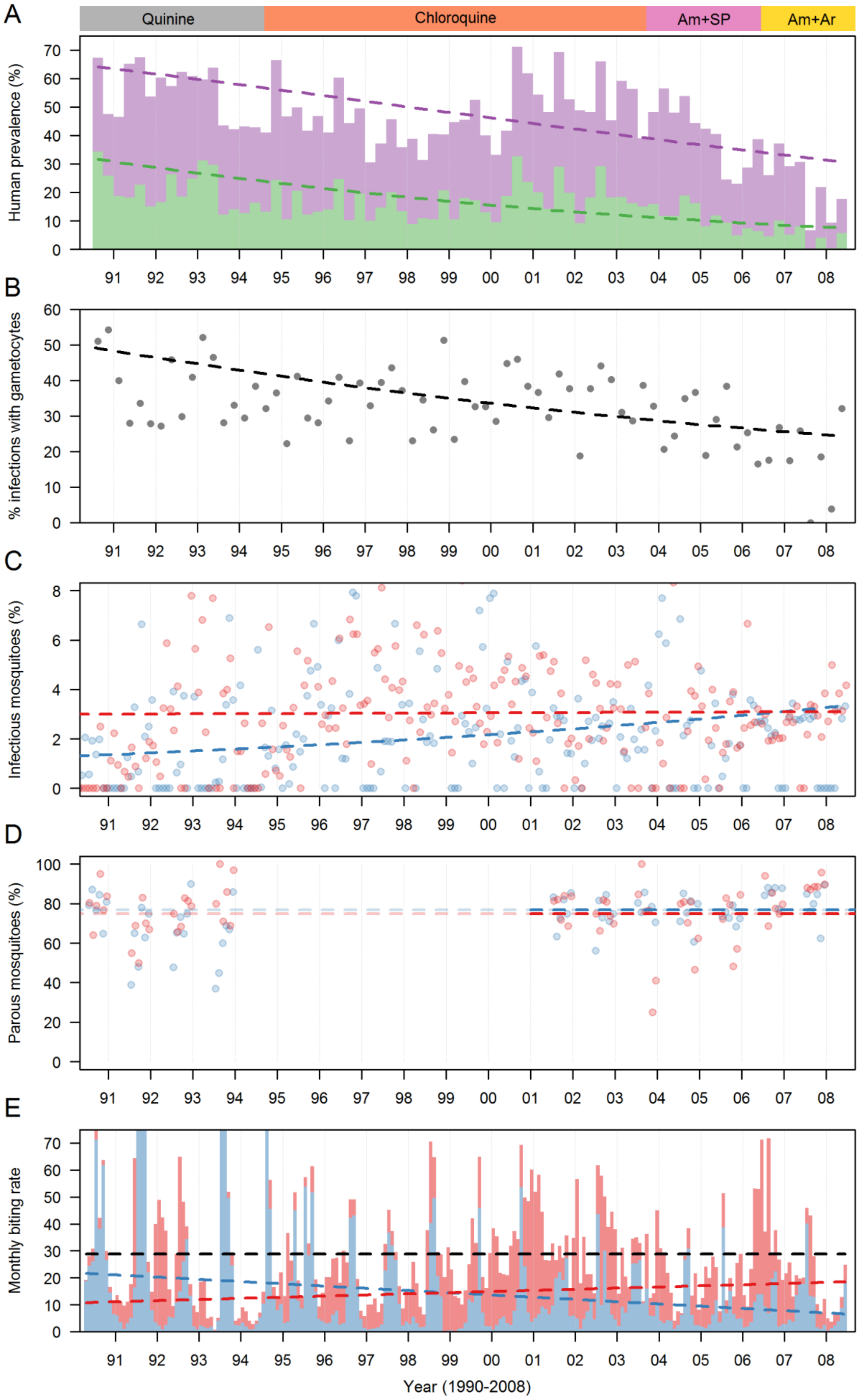

Supplementary Figure 1. Temporal analysis of the Dielmo data between 1990 and July 2008. (A) The changes in *Plasmodium falciparum* prevalence of asexual parasites (purple) and gametocytes (green) in the enrolled

population as measured by microscopy. (B) The percentage of slide positive patients with detectable gametocytes. (C) The percentage of *Anopheles gambiae s.l.* (blue) and *A. funestus* (red) with salivary gland sporozoites. (D) The parity of *A. gambiae s.l.* (blue) and *A. funestus* (red). (E) The cumulative monthly biting rate of *A. gambiae s.l.* (blue) and *A. funestus* (red). In all panels the overall trend over the time period is shown by the dashed lines (the best fit model of mixed-effects logistical regression in A-D and mixed-effects linear regression in E). The changes in the drugs used for first line therapy are shown at the top of the figure, be it quinine (grey), chloroquine (orange), amodiaquine plus sulphadoxine-pyrimethamine (Am+SP, pink) or amodiaquine plus artesunate (Am+Ar, yellow). Over the study period logistic regression indicates a significant reduction in asexual (p<0.0001) and gametocyte (p<0.0001) slide prevalence (A). Gametocyte slide prevalence falls at a significantly faster rate than asexual slide prevalence (p=<0.0001,B). There is no evidence of a change in the proportion of infectious *A. funestus* between 1990 and 2008 (p=0.782) though there is a significant increase in the proportion of infected *A. gambiae* (p<0.0001,C). Information on the number of mosquitoes dissected was only available between 2001-2008 so statistical analysis was restricted to this period (though a lighter version of the line is included for the whole period for visual comparison). There was no evidence in a temporal trend in parity between 2001 and 2008 (either in *A. funestus, p*= 0.885, or *A. gambiae, p*=0.136). There was also no overall trend in the total human biting rate between 1990 and 2008 (p=0.068) though there is a significant increase in the number of *A. funestus* caught (p=0.0076) whilst the number of *A. gambiae s.l.* significantly declined over the period (p<0.0001). Though the species composition has changed this does not explain the persistence of mosquito infection as the percentage of infectious mosquitoes will be independent of their relative abundance.

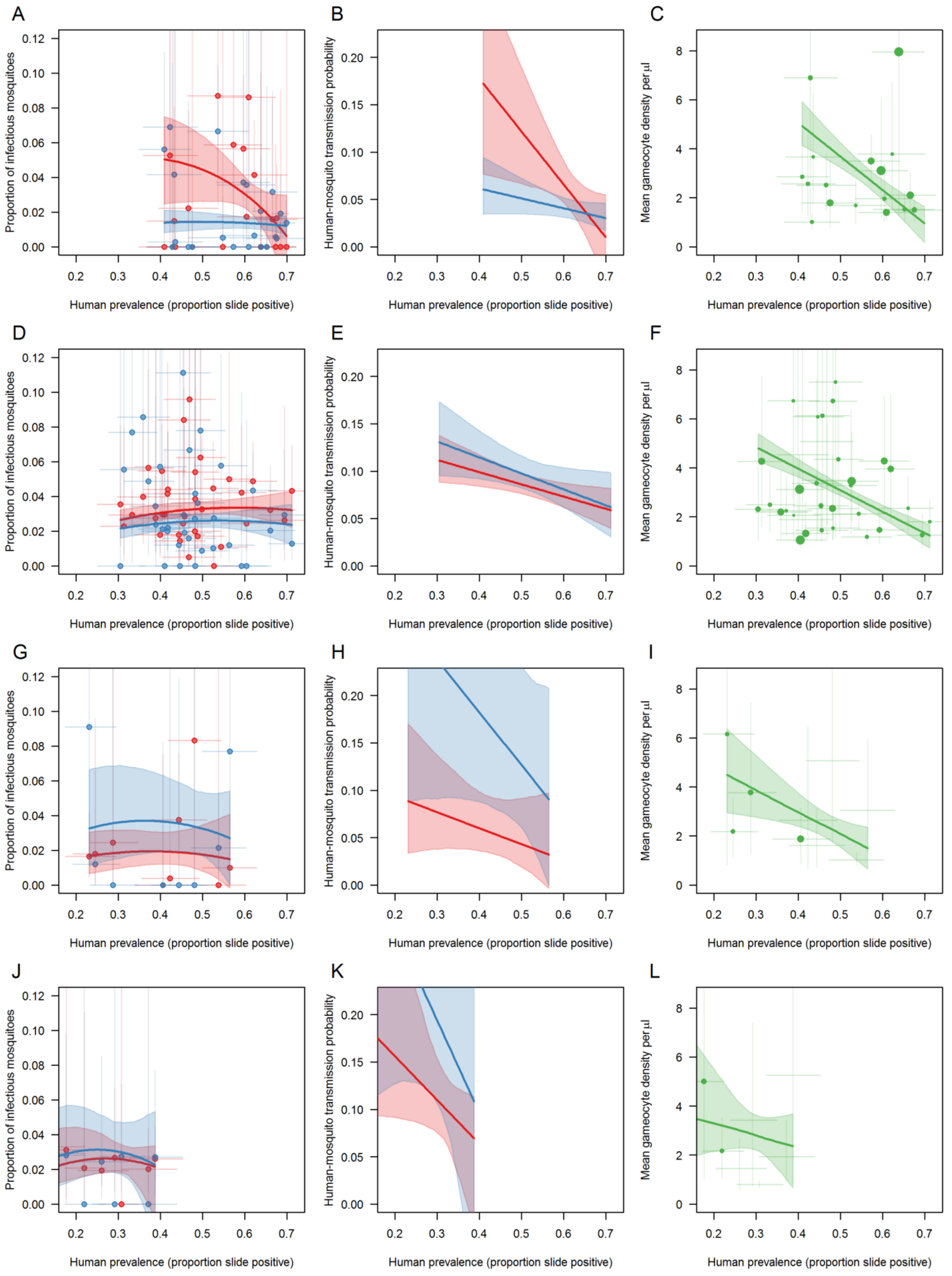

Supplementary Figure 2. Verification that the observed trends in transmission probability is independent of frontline therapy. The full dataset outlined in Supporting Information 3 is divid

ed into periods with quinine as frontline treatment of malaria cases (panels A-C), chloroquine (panels D-F), amodiaquine plus sulphadoxine-pyrimethamine (G-I) or amodiaquine plus artesunate (J-L). Models outlined in the main text were rerun allowing the human-mosquito transmission probability (the parameters that make

up function *c*, see equation 2 of the main text) to vary between time periods. Panels (A,D,G,J) show the relationship between the prevalence of malaria in humans and mosquitoes. Each point shows human and entomological surveys conducted during the same month. Blue points are *Anopheles gambae s.l.* and red *A. funestus*. Thick lines give the best fit model for each mosquito species. Panels B,E,H,K show the change in transmission efficiency over the period. (C,F,I,L) show gametocyte density in gametocyte positive patients as detected by microscopy. In all plots horizontal and vertical lines denote the 95% confidence intervals around point estimates whilst shaded areas show model uncertainty.

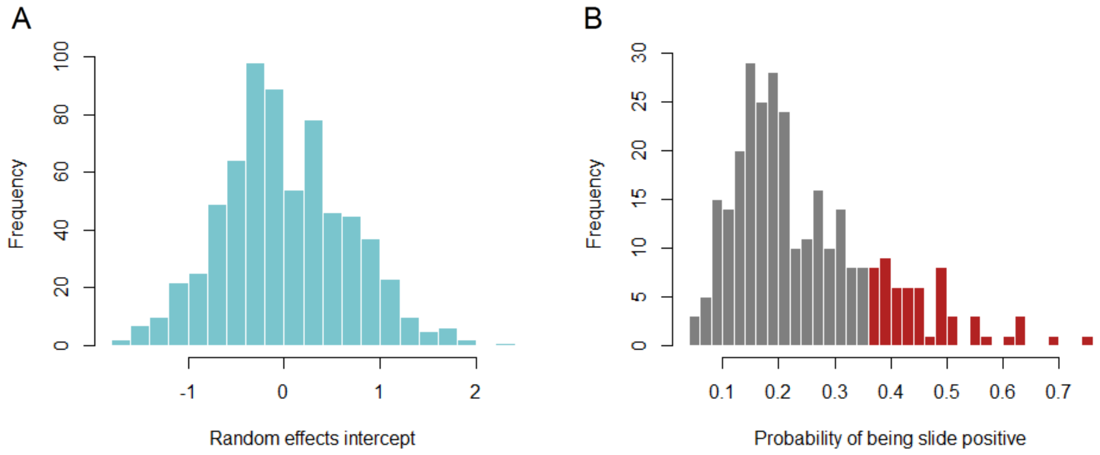

Supplementary Figure 3. The variability in probability of having detectable asexual parasites between patients as estimated by a mixed-effects logistic regression. (A) Frequency distribution of the individual patient random effects estimates (intercepts) on the logit scale (standard deviation = 0.86). (B) Model predictions of the variability in the probability of being slide positive for a patient in the 10-14 age group in the last quarter of 2007. Red bars highlight the 20% of the population which the model predicts will have the highest probability of being slide positive over the course of the study. Collectively, at this time point, the model predicts that these individuals would account for only 37% of all positive infections. A much larger percentage would be expected if these hosts were repeatedly being identified as having asexual parasites. In (A) all patients included in the study whilst (B) shows those who were sampled in the last quarter of 2007. The fixed effect time coefficients of the model are given in Supplementary Table 2 whilst the coefficients of the different age groups is as follows (2-4 years=0.70, 5-9 years =0.87, 10-14 years =0.91, 15-29 years =0.48, 30-44=-0.24 and >44 years =-0.60).

	Constant	Linear	Polynomial	Best fit parameters
**A). Asexual parasites prevalence**				
Single relationship for all mosquito species	1364.0	1325	1327	α_1^g =0.22 (0.11, 0.19) α_2^g =-0.27 (-0.36,-0.18) α_1^f =0.15 (0.11, 0.19) α_2^f =-0.13 (-0.21, -0.06)
Different relationship for the two mosquito species	1365.0	1323*	1324	
Different relationship + variable mortality rate	1365	1328	1330	
**B). Gametocyte prevalence**				
Single relationship for all mosquito species	1330	1260	1254	α_1^g =0.22 (0.11,0.19), α_2^g =-0.27 (-0.36,-0.18), α_3^g =-0.27 (-0.36,-0.18), α_1^f =0.15 (0.11,0.19), α_2^f =-0.13 (-0.21,-0.06), α_3^f =-0.13 (-0.21,-0.06),
Different relationship for the two mosquito species	1328	1254	1234*	
Different relationship + variable mortality rate	1328	1254	1236	
**C). Gametocyte density in gametocyte positive individuals**				
Density independent of therapy	15830	15780	15770	α_1 =10.2 (0.0,39), α_2 = -0.23 (-95,52), α_3 =-14.1 (-71,63), k =0.64 (0.61,0.67)

Supplementary Table 1. The goodness of fit for the different models. A) Relationship between human asexual parasite prevalence and sporozoite prevalence in mosquitoes. B). Relationship between human gametocyte prevalence and sporozoite prevalence in mosquitoes. C). Relationship between asexual parasite prevalence and gametocyte density in gametocyte positive individuals. The lower the Deviance Information Criterion the more parsimonious the model. The * signifies best fit model whose parameters are given (including 95% Bayesian credibility intervals). Superscript differentiates mosquito species, be it *Anopheles gambiae s.l.* (α_1^g) or *A. funestus* (α_1^f). Parameter k in C gives the overdispersion parameter of the negative binomial distribution. DIC values for the different datasets (A,B and C) are not directly comparable.

Year	Time-points	Frontline treatment	Mean nights collection	Mean number samples per time point			Model coefficient
				Humans	*A. gambiae*	*A. funestus*	
1990	6	Quinine	11 (8,12)	1530 (159,2294)	438 (172,854)	44 (22,99)	-
1991	4	Quinine	12 (12,12)	193 (181,204)	332 (38,983)	152 (47,375)	-0.34, 0.88, 1.01, 0.28
1992	4	Quinine	12 (12,12)	199 (151,225)	107 (24,313)	242 (51,558)	0.65, 0.48, 0.74, 0.67
1993	4	Quinine	12 (12,12)	240 (237,249)	293 (5,1112)	55 (2,195)	0.60, 0.81, -0.16, -0.26
1994	4	Quinine	12 (12,12)	246 (236,259)	69 (24,125)	50 (15,67)	-0.19, -0.21 -0.33, 0.92
1995	4	Chloroquine	18 (16,20)	260 (245,273)	205 (63,452)	125 (72,200)	-0.06, 0.09, -0.36, -0.07
1996	4	Chloroquine	16 (16,16)	281 (271,293)	212 (42,674)	169 (48,270)	-0.42, 0.56, -0.21, 0.03
1997	4	Chloroquine	18 (16,20)	254 (240,269)	186 (18,641)	124 (106,141)	-0.99, -0.64 -0.25, -0.70
1998	4	Chloroquine	19 (16,20)	275 (258,300)	279 (1,993)	280 (89,402)	-0.58, -0.92 -0.44, -0.45
1999	4	Chloroquine	18 (16,20)	267 (253,280)	101 (3,298)	257 (208,359)	-0.23, -0.18 0.16, -0.47
2000	4	Chloroquine	19 (16,20)	282 (274,295)	226 (13,484)	471 (250,752)	-0.80, -0.33 1.17, 0.66
2001	4	Chloroquine	15 (12,20)	268 (247,309)	216 (105,327)	352 (101,826)	0.29, 0.03, 1.04, 0.14
2002	4	Chloroquine	10 (4,16)	250 (236,270)	100 (4,190)	326 (37,767)	0.03, 0.00, 0.90, -0.20
2003	4	Chloroquine	10 (4,16)	260 (239,279)	70 (24,178)	77 (3,191)	0.53, 0.41, -0.44, -0.03
2004	4	Am+SP	8 (4,12)	241 (228,256)	92 (4,320)	90 (12,231)	0.39, 0.01, 0.28, -0.23
2005	4	Am+SP	12 (12,12)	219 (204,245)	49 (14,83)	157 (48,275)	-0.40, -0.41 -1.28, -1.44
2006	4	Am+SP	9 (4,12)	234 (219,261)	56 (6,111)	396 (48,778)	-1.03, -0.49 -1.05, -0.68
2007	4	Am+Ar	8 (8,8)	223 (30,297)	70 (8,145)	83 (13,150)	-0.97, -0.86 -2.45, -1.34
2008	2	Am+Ar	8 (8,8)	299 (280,318)	41 (10,71)	26 (20,32)	-2.46, -1.68

Supplementary Table 2. Summarising the data used to fit the relationship between malaria slide prevalence and the proportion of infectious mosquitoes. Values in brackets give the range. Model coefficients give the best fit estimate (in order,on the logit scale) for the mixed-effect logistic regression presented in Supplementary Fig.3. It was not possible to generate coefficient estimates for 1990 due to an alternative patient coding which made the 1st quarter of 1991 the model intercept.
